# Editorial on special issue “Metamaterials and Plasmonics in Asia”

**DOI:** 10.1515/nanoph-2024-0101

**Published:** 2024-03-18

**Authors:** Takuo Tanaka, Lei Zhou, Q-Hang Park, Atsushi Sanada

**Affiliations:** Metamaterials Lab., RIKEN, 2-1 Hirosawa, 351-0198, Wako, Saitama, Japan; Surface Physics Laboratory and Physics Department, Fudan University, Shanghai 200433, Shanghai, China; Department of Physics, 34973 Korea University, 02841, Seoul, South Korea; 13013 Osaka University, Toyonaka, Osaka, Japan

Metamaterials and plasmonics are the most important research fields in the nanophotonics community. These fields have gained significant prominence since the beginning of this century. In the early stages of metamaterials research, there was intensive study and discussion on negative refraction and negative index materials. Metamaterials provide an excellent platform to realize such optical phenomena. While metamaterials research initially focused on the microwave frequency region, it has since then been extended to the entire optical range, from THz waves to ultraviolet. Moreover, the concept of metamaterials has been expanded beyond electromagnetics to encompass many fields that utilize waves.

**Figure 1:**
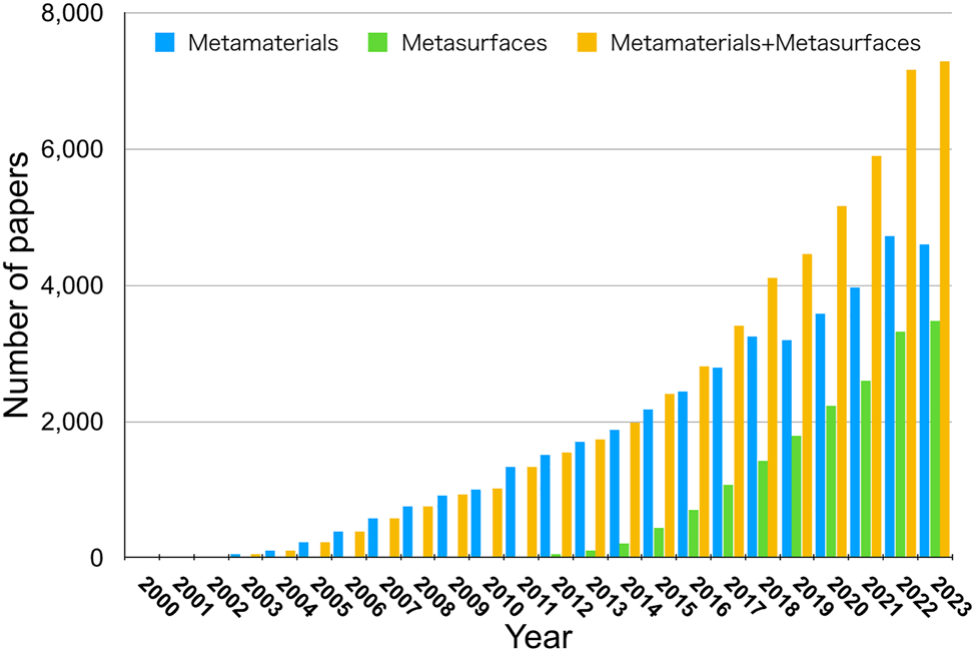
Trend in the number of publications on metamaterials research.

Figure 1 shows the trend in the number of publications of journal papers on metamaterials research. Despite the COVID-19 pandemic, the number has increased monotonically, with more than 7000 papers published in 2023. In 2011, Capasso proposed the concept of “metasurfaces,” which are 2D versions of metamaterials [1]. His paper, published in Science, opened up new trends in metamaterial research. In fact, the number of papers related to metasurfaces has dramatically increased since 2011, and now the number is almost equivalent to that of metamaterials.

This special issue, “Metamaterials and Plasmonics in Asia,” was developed from the A3 Metamaterials Forum held from June 26th to 29th, 2023, at Kyoto Institute of Technology in Kyoto, Japan. The A3 Metamaterials Forum is an annual meeting that discusses cutting-edge research results in metamaterials and plasmonics among three Asian countries: Korea, Japan, and China.

Five review articles are presented in this special issue. Zhang et al. provide an overview of recent progresses in programmable optical meta-holograms and provide outlook on the challenges and prospects in this growing area [2]. Deng et al. summarize inverse design techniques for photonic crystal structures [3]. Fu et al. discuss the contribution of the machine learning techniques to advanced metasurface research [4]. Chu et al. review recent advances in thermal emission control by metasurface technologies and propose their infrared applications, such as infrared sensing, radiative cooling, and thermophotovoltaic devices [5]. Ding et al. review spectral imaging techniques using metasurface devices [6].

In addition to these review articles, this issue also contains 20 original papers. Kang et al. propose an ultrafast imaging technique of terahertz electric potentials across ring-shaped quantum barriers [7]. Shi et al. propose a zoom metalens fabricated on a flexible thin PDMS film. By stretching the substrate, focal length of the metalens could be changed and a 2× range of free magnification control was demonstrated [8]. Zhu et al. develop a spatiotemporal metasurface for beamforming applications, eliminating the Doppler effect of fast-moving automobiles, trains, aircrafts, and so on, using tandem neural network system [9]. Nakayama et al. report on a metasurface absorber for enhancing the conversion efficiency in the thermoelectric device of BiSbTe [10]. Sang et al. propose the concept of relative shift-induced quasi-bound states in the continuum and demonstrate asymmetry parameter-insensitive resonant modes using an array of silicon square disk dimers [11]. Xue et al. demonstrate a beam shaper metasurface doublet that converts a Gaussian beam into a flat-top beam using the complex-amplitude constraint Gerchberg–Saxton algorithm [12]. Guo et al. discuss the robustness of an integrated topological interface of valley photonic crystals against sharp bending [13]. Yan et al. report on-chip transmissible topological edge states using one-dimensional Su–Shrieffer–Heeger photonic crystals with defect cavities on a silicon-on-insulator slab and demonstrate wavelength division multiplexer devices [14]. Hong et al. also report a color router metasurface that can split the spectrum from visible to near-infrared and redirect it to the four optical channels (red, green, blue, and near-infrared) of a CMOS image sensor [15]. Lee et al. propose a deterministic reflection contrast ellipsometry technique for multilayer 2D heterostructures [16]. Yamaguchi et al. demonstrate a high-resolution (2322 × 2322 pixels) and full-color holographic movie using silicon nitride metasurface [17]. Ikeda et al. discuss the spatial and temporal properties of emission enhancement in InGaN/GaN by surface plasmon resonance [18]. Liu et al. introduce the diffractive neural network technique into a dielectric metasurface made of silicon and applied it to a 1 bit adder as an optical computing component [19]. Kim and Park demonstrate a perfect waveguide coupler with universal impedance matching [20]. Kim et al. propose the realization of two-dimensional Bravais lattice patterns formed by a metasurface-based interference lithography technique [21]. Lee et al. discuss the contribution of the hBN layers to the enhancement of the guided exciton–polariton modes in multilayer waveguides of WS_2_ [22]. Park et al. propose a sample-efficient inverse design algorithm of freeform nanophotonic devices using a physics-informed reinforcement learning technique [23]. Dai et al. report spintronic terahertz emission from a metasurface using scanning near-field nanoscopy [24]. Liu et al. propose thin film electro-optic modulator using lithium niobate [25]. Zhang et al. demonstrate holographic communication using programmable metasurafces [26].

We, symposium organizers, believe that this special issue provides a comprehensive overview of recent research activities by leading scientists in the field of metamaterials and plasmonics in Asia. Finally, we sincerely appreciate all contributions from the authors to this special issue.
